# Detection of hypermethylated genes as markers for cervical screening in women living with HIV

**DOI:** 10.1002/jia2.25165

**Published:** 2018-08-13

**Authors:** Wieke W Kremer, Marjolein Van Zummeren, Putri W Novianti, Karin L Richter, Wina Verlaat, Peter JF Snijders, Daniëlle AM Heideman, Renske DM Steenbergen, Greta Dreyer, Chris JLM Meijer

**Affiliations:** ^1^ Department of Pathology Cancer Center Amsterdam VU University Medical Center Amsterdam The Netherlands; ^2^ Department of Epidemiology and Biostatistics VU University Medical Center Amsterdam The Netherlands; ^3^ Department of Medical Virology University of Pretoria and National Health Laboratory Services Pretoria South Africa; ^4^ Department of Obstetrics and Gynaecology University of Pretoria Pretoria South Africa

**Keywords:** DNA Methylation Marker Testing, Early Detection of Cancer, Human Immuno‐deficiency Virus, Human Papillomavirus, High‐grade Cervical Intraepithelial Neoplasia, Uterine Cervical Neoplasms

## Abstract

**Introduction:**

To evaluate the performance of hypermethylation analysis of *ASCL1*,*LHX8* and *ST6GALNAC5* in physician‐taken cervical scrapes for detection of cervical cancer and cervical intraepithelial neoplasia (CIN) grade 3 in women living with HIV (WLHIV) in South Africa.

**Methods:**

Samples from a prospective observational cohort study were used for these analyses. Two cohorts were included: a cohort of WLHIV who were invited for cervical screening (*n *=* *321) and a gynaecologic outpatient cohort of women referred for evaluation of abnormal cytology or biopsy proven cervical cancer (*n *=* *108, 60% HIV seropositive). Cervical scrapes collected from all subjects were analysed for hypermethylation of *ASCL1*,*LHX8* and *ST6GALNAC5* by multiplex quantitative methylation specific PCR (qMSP). Histology endpoints were available for all study subjects.

**Results:**

Hypermethylation levels of *ASCL1*,*LHX8* and *ST6GALNAC5* increased with severity of cervical disease. The performance for detection of CIN3 or worse (CIN3^+^) as assessed by the area under the receiver operating characteristic (ROC) curves (AUC) was good for *ASCL1* and *LHX8* (AUC 0.79 and 0.81 respectively), and moderate for *ST6GALNAC5* (AUC 0.71). At a threshold corresponding to 75% specificity, CIN3^+^ sensitivity was 72.1% for *ASCL1* and 73.8% for *LHX8* and all samples from women with cervical cancer scored positive for these two markers.

**Conclusions:**

Hypermethylation analysis of *ASCL1* or *LHX8* in cervical scrape material of WLHIV detects all cervical carcinomas with an acceptable sensitivity and good specificity for CIN3^+^, warranting further exploration of these methylation markers as a stand‐alone test for cervical screening in low‐resource settings.

## 1 Introduction

Women living with human immunodeficiency virus (WLHIV) have an increased risk for the development of cervical cancer and its precursor lesions, classified as cervical intraepithelial neoplasia (CIN) grade 1 to 3 1, 2, 3. Compared to HIV uninfected women, WLHIV develop cervical cancer at a younger age and are more likely to die of the disease 4, 5, 6. Both HIV and cervical cancer have a disproportionally high burden in low‐ and middle‐income countries (LMIC): more than 95% of global HIV infections and more than 85% of all cervical cancer cases occur in less developed regions 7, 8. Here, cervical cancer is a leading cause of cancer‐related death in women, partly caused by the high incidence of HIV, but also caused by the absence of effective cervical screening programmes and limited access to healthcare 8. Implementation of screening in low‐resource settings is challenging and the development of screening methods that are suitable for this setting is warranted.

Cervical cancer and CIN are caused by a persistent infection with high‐risk human papillomavirus (hrHPV) 9, 10. Primary hrHPV testing is currently the preferred method for cervical screening, irrespective of resource settings or HIV‐prevalence 11. However as most infections are self‐limiting and do not cause cervical lesions, hrHPV testing has limited specificity 12, 13, particularly in WLHIV 14, 15, 16. Therefore, subsequent triage testing of hrHPV positive women is needed to distinguish women with underlying high‐grade cervical disease from women with transient infections 17, 18. Available triage tests for LMIC recommended by the World Health Organization (WHO) include cytology, partial hrHPV genotyping or visual inspection with acetic acid (VIA) 19. Major limitations of these two‐step approaches include the risk of loss to follow‐up and their requirement of technical capabilities and healthcare infrastructure, specifically relevant in low‐resource settings 20. In addition, in a setting with a high HPV prevalence, triage strategies require a large number of tests. A single and objective point‐of‐care test with a high sensitivity and specificity for CIN3 and cervical cancer in both HIV seropositive and HIV seronegative women overcomes these limitations and would be most effective to improve cervical screening in LMIC.

A candidate primary test to identify women at risk for clinically meaningful cervical disease is hypermethylation analysis of promoter regions of host cell genes involved in cervical carcinogenesis 21, 22, 23. Hypermethylation of gene promotor regions results in gene silencing and represents an essential step for cervical cancer development 21, 24. Assays detecting hypermethylation are objective and can be applied on various specimen types, including self‐collected cervical material 25, 26, 27. Multiple genes have been identified as possible targets for cervical precancer and cancer detection, but few have been evaluated in WLHIV 14, 28, 29, 30. We previously showed that methylation analysis of *CADM1*,* MAL* and *miR124‐2* genes in cervical scrapes from hrHPV positive WLHIV is an acceptable triage tool, which detects all cervical carcinomas and the majority of CIN3 14. However, specificity of this marker panel when evaluated as a primary screening tool, without prior hrHPV testing, was limited. Therefore, additional markers that can be used for cervical screening in WLHIV in low‐resource settings without prior hrHPV testing should be evaluated.

In a recent genome‐wide DNA methylation profiling study, three hypermethylated genes, Achaete‐scute Family bHLH Transcription Factor 1 (*ASCL1*), LIM Homeobox 8 (*LHX8*) and ST6 N‐Acetylgalactosaminide Alpha‐2,6‐Sialyltransferase 5 (*ST6GALNAC5*), were identified as promising triage markers in hrHPV positive women for cervical cancer and CIN3 31. The present study evaluates the performance of these methylation markers for the detection of CIN3 and cervical cancer in WLHIV irrespective of their HPV status.

## 2 Methods

### 2.1 Study population and procedures

**Figure 1:**
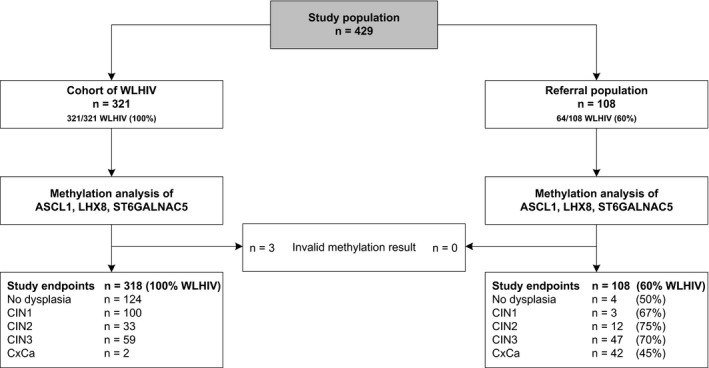
Study flow chart. CIN, cervical intraepithelial neoplasia; CxCa, cervical carcinomas; WLHIV, women living with HIV.

The study population is outlined in Figure 1 and consists of two groups: a cohort of WLHIV and a gynaecological referral population. The total study population included 429 women who were originally included between February 2013 and March 2015 at a gynaecologic outpatient clinic in Steve Biko Academic Hospital or Tshwane District Hospital, Pretoria, South Africa, in a study (Ethical Committee of the University of Pretoria, South Africa protocol numbers 100/2012 and 155/2014) comparing different cervical screening strategies. These women previously had a valid study endpoint and were eligible for inclusion in the present report. All participants were aged 18 years and above, and had not been treated for cervical cancer or precancer in the preceding two years. Nearly all women (99%) in the cohort of WLHIV were on antiretroviral treatment (ART) and their median CD4^+^ cell count was 514 cells/μL. In total, 60% of women in the referral cohort were HIV seropositive of whom 37% were on ART; their median CD4^+^ cell count was 342 cells/μL. High‐risk HPV positivity was 42% in the cohort of WLHIV and 95% of women in the referral population. Median age was 41 [interquartile range (IQR): 35 to 46 years] in the cohort of WLHIV and 44 years (IQR: 34 to 51 years) in the referral population. Detailed characteristics, inclusion criteria and study procedures of this study have been described previously 14. Written informed consent was obtained from all participants.

In short, HIV‐infected women visiting the gynaecologic outpatient clinic for cervical screening were included in the cohort of WLHIV. Cervical cells were collected using a Cervex Brush^®^ (Rovers Medical Devices B.V., Oss, the Netherlands) and, after preparation of a conventional slide, stored in Thinprep PreservCyt^®^ solution (Hologic, Marlborough, MA, USA). Colposcopy was performed for all participants and two biopsies from either the most severe cervical lesion or, if no lesion was present, two random biopsies were collected. The referral population included women who visited the gynaecologic outpatient department for evaluation of an abnormal Pap smear [≥high‐grade squamous intraepithelial neoplasia (HSIL)] or biopsy‐proven cervical cancer. A cervical scrape was also collected from these women and the material was stored in Thinprep PreservCyt solution.

Women with abnormal cytology (≥HSIL) or CIN2 or worse (CIN2^+^) on a cervical biopsy received treatment according to local guidelines [large loop excision of the transformation zone (LLETZ) or clinical cancer staging]. Study endpoints were based on histological diagnosis of either the cervical biopsy or LLETZ specimen (worst histology). Participants with invalid histology results were excluded from the analysis.

### 2.2 Reference population

Cervical scrapes from a Dutch reference population (*n *=* *265; study endpoints: 196 ≤ CIN1, 30 CIN3, 39 cervical carcinomas; median age 40 years (IQR: 32 to 47 years)) were used only to compare methylation levels between HIV seronegative and HIV seropositive women (manuscript in preparation). This study group was assumed to be HIV seronegative, since HIV incidence rates in the Netherlands are very low (0.1% in women) 32.

### 2.3 Methylation analysis

For methylation analysis, DNA previously isolated from cervical scrapes was bisulphite‐converted using the EZ DNA Methylation Kit (Zymo Research, Irvine, CA, USA). Multiplex quantitative methylation‐specific PCR (qMSP) for *ASCL1*,* LHX8* and *ST6GALNAC5* was performed as described previously using 50 ng of bisulphite‐converted DNA, EpiTect MethyLite Master Mix (Qiagen, Hilden, Germany) and 100 to 300 nmol/L of each primer and fluorescent dye‐labelled probe 31. Housekeeping gene β‐actin (ACTB) was used as a reference to assure sample quality and successful bisulphite conversion. A plasmid containing the amplicon sequences of all targets and ACTB was used as a calibrator. Cycle threshold (Ct) values were measured at a fixed fluorescence threshold. Methylation values of all targets were normalized to the reference gene and the calibrator using the comparative Ct method (2^−∆∆Ct^ × 100) to obtain ∆∆Ct ratios 33. Analyses were done on the ViiA 7 real‐time PCR system (Applied Biosystems, Foster City, CA, USA). All samples with ACTB Ct ratios >30 were considered invalid and were excluded from the analysis (*n *=* *3).

### 2.4 Statistical analysis

The Kruskal–Wallis omnibus test was performed on each methylated gene to assess differences in methylation levels among disease categories. Following a significant result from the omnibus test, post‐hoc testing was then performed using Mann–Whitney *U*. Bonferroni correction was subsequently used to correct *p*‐values for multiple testing. Log10‐transformed Ct ratios were visualized in boxplots for the cohort of WLHIV and the referral population together. To assess the effect of HIV status on methylation levels of *ASCL1*,* LHX8* and *ST6GALNAC5*, differences in methylation levels between HIV seropositive and HIV seronegative women within cases [CIN3 or worse (CIN3^+^)] and controls [CIN1 or less (≤CIN1)] were calculated using Mann–Whitney U. For this analysis, the Dutch reference population was used to enrich the group of HIV seronegative samples.

To assess the performance of each individual methylation marker to distinguish cases from controls in cervical scrapes from women from the cohort of WLHIV, univariable logistic regression analysis was performed on the square root transformed Ct ratios. Histologically classified CIN2 is a heterogeneous group of cervical disease which can be either the results of a productive or transforming HPV infection 22. Therefore, samples from women with CIN2 were excluded from this analysis.

Then, the clinical performance of each individual marker to detect CIN3^+^ in the cohort of WLHIV was evaluated by the leave‐one‐out cross‐validation approach. With this approach, predicted probabilities were calculated for each sample, representing the risk for an underlying CIN3^+^. Receiver operating characteristic (ROC) curves from the cross‐validated predicted probabilities were used to visualize the performance of the logistic regression models and were evaluated by area under the ROC curve (AUC). Based on the ROC curves, fixed thresholds for predicted probabilities corresponding to 75% and 80% specificity were chosen and corresponding CIN3^+^ sensitivities were calculated. In addition, positivity rates for CIN2 were calculated.

To further evaluate the clinical performance for detection of CIN3 and cancer, the logistic regression models described above were applied in the referral population. Positivity rates per disease category were calculated for each methylation marker using the fixed thresholds.

All calculations were performed in Microsoft Excel (2010), SPSS (V. 22), R (V. 3.3.1) and GraphPad Prism (V 7.02).

## 3 Results

Four hundred and twenty‐six women from the South African study cohorts were included in this report. An overview of the histology endpoints is given in Figure 1.

### 3.1 Methylation levels across disease categories and HIV status

**Figure 2:**
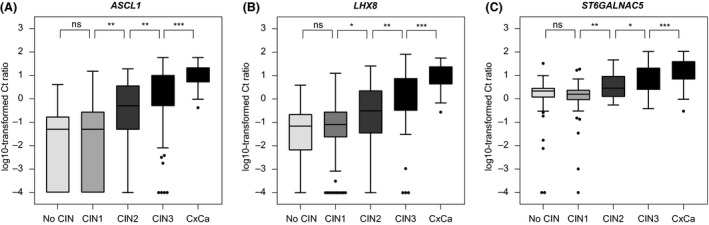
Methylation levels increase with severity of underlying cervical disease. Methylation levels of *ASCL1* **(A)**,* LHX8* **(B)** and *ST6GALNAC5* **(C)** represented by the log10‐transformed Ct ratios (*y*‐axis) in the different histology subgroups (*x*‐axis) from the cohort of WLHIV and the referral population combined. CIN, cervical intraepithelial neoplasia; CxCa, cervical carcinomas. *p < 0.05; **p < 0.01; ***p < 0.001; ns, not significant.

Differences across cervical disease categories in methylation levels of *ASCL1*,* LHX8* and *ST6GALNAC5* were evaluated in the two South African study cohorts combined. As shown in Figure 2, methylation levels of *ASCL1* (Figure 2A), *LHX8* (Figure 2B) and *ST6GALNAC5* (Figure 2C) increased significantly with severity of the underlying cervical lesion.

**Figure 3:**
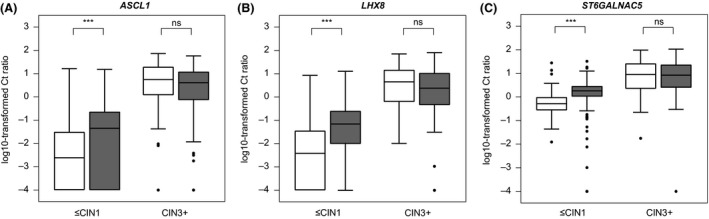
Comparing methylation levels between HIV seronegative and HIV seropositive women. Methylation levels of *ASCL1* **(A)**,* LHX8* **(B)** and *ST6GALNAC5* **(C)** in cervical scrapes from HIV seronegative (white) and HIV seropositive (grey) women. The *y*‐axis displays the log10‐transformed Ct ratios. The *x*‐axis displays the histology subgroups for HIV seronegative and HIV seropositive women. CIN, cervical intraepithelial neoplasia. ***p < 0.001; ns, not significant.

To assess a potential influence of HIV status on methylation levels of *ASCL1*,* LHX8* and *ST6GALNAC5*, the Ct ratios of each marker were stratified by HIV status and compared within cases (CIN3^+^) and controls (≤CIN1) using the Dutch reference population. Five women from the South African population with unknown HIV status were excluded from this analysis. Median age of HIV seropositive women and HIV seronegative women did not differ. Methylation levels of *ASCL1* (Figure 3A), *LHX8* (Figure 3B) and *ST6GALNAC5* (Figure 3C) were significantly higher in HIV seropositive women with ≤CIN1, compared to HIV seronegative women with ≤CIN1 (*p* < 0.001). In women with CIN3^+^ methylation levels were comparable between HIV seropositive and HIV seronegative women (*p* > 0.05).

### 3.2 Performance of primary methylation marker analysis

To determine the clinical performance of *ASCL1*,* LHX8* and *ST6GALNAC5* to distinguish cases (*n = *61) from controls (*n = *224), logistic regression analysis was performed in the South African cohort of WLHIV. All three methylation markers significantly distinguished cases from controls (*p* < 0.001).

**Figure 4:**
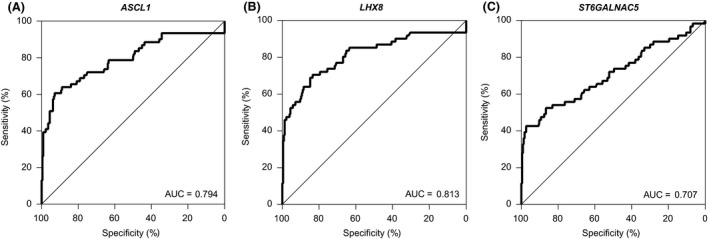
Individual marker performance for CIN3^+^ detection. ROC curves from the cross‐validated predicted probabilities of *ASCL1* **(A)**,* LHX8* **(B)** and *ST6GALNAC5* **(C)** for CIN3^+^ detection in cervical scrapes from WLHIV (cohort of WLHIV only). ROC, receiver operating characteristics; AUC, area under the ROC curves.

Subsequently, the clinical performance of *ASCL1*,* LHX8* and *ST6GALNAC5* to detect CIN3^+^ was evaluated by leave‐one‐out cross‐validation approach. *ASCL1* and *LHX8* showed a good clinical performance, visualized by ROC curves and quantified by AUCs being 0.79 for *ASCL1* (Figure 4A) and 0.81 for *LHX8* (Figure 4B). *ST6GALNAC5* showed a moderate performance with an AUC of 0.71 (Figure 4C). Based on these ROCs, fixed thresholds corresponding to a specificity of 75% and 80% were chosen. At fixed thresholds corresponding to 75% specificity, the sensitivity for CIN3^+^ was 72.1% (95% confidence interval (CI) 59.2 to 82.9) for *ASCL1*, 73.8% (95%CI 60.9 to 84.2) for *LHX8* and 55.7% (95%CI 42.4 to 68.5) for *ST6GALNAC5* (Table 1). Positivity rates for CIN2 at these thresholds were 48.5% (16/33) for *ASCL1*, 42.4% (14/33) for *LHX8* and 33.3% (11/33) for *ST6GALNAC5*. At fixed thresholds corresponding to 80% specificity, the values of sensitivity were slightly reduced, resulting in 67.2% (95%CI 57.4 to 78.7) sensitivity for *ASCL1*, 70.5% (95%CI 57.4 to 81.5) for *LHX8* and 54.1% (95%CI 40.8 to 66.9) for *ST6GALNAC5*. Positivity for CIN2 at these thresholds were 42.4% (14/33) for *ASCL1*, 42.4% (14/33) for *LHX8* and 33.3% (11/33) for *ST6GALNAC5*.

**Table 1:** Performance of *ASCL1*,* LHX8* and *ST6GALNAC5* at fixed thresholds for 75% and 80% specificity

	75% specificity				80% specificity			
	CIN3^+^ sensitivity	95%CI	*n*1/*N*1	positivity rate in CIN2	CIN3^+^ sensitivity	95%CI	*n*1/*N*1	positivity rate in CIN2
Cohort of WLHIV (*n* = 318)								
*ASCL1*	72.1%^a^	59.2 to 82.9	44/61	48.5%	67.2%^a^	54.0 to 78.7	41/61	42.4%
*LHX8*	73.8%^a^	60.9 to 84.2	45/61	42.4%	70.5%	57.4 to 81.5	18/61	42.4%
*ST6GALNAC5*	55.7%	42.4 to 68.5	34/61	33.3%	54.1%	40.8 to 66.9	28/61	33.0%
Referral cohort (*n *=* *108)								
*ASCL1*	94.4%^a^	89.6 to 99.2	84/89	75.0%	94.4%^a^	89.6 to 99.2	84/89	75.0%
*LHX8*	92.1%^a^	86.5 to 97.7	82/89	75.0%	89.9%	83.6 to 97.7	80/89	75.0%
*ST6GALNAC5*	89.9%	83.6 to 97.7	80/89	83.3%	89.9%	83.6 to 97.7	80/89	83.3%

Performance of *ASCL1*,* LHX8* and *ST6GALNAC5* for the detection of cervical intraepithelial neoplasia 3 or worse (CIN3^+^). CI, confidence interval; CIN, cervical intraepithelial neoplasia; n1, number of test positive disease cases; N1, total number of disease cases; WLHIV, women living with human immunodeficiency virus; ^a^, all carcinomas were positive at this threshold.

### 3.3 Methylation positivity in a gynaecological referral population

To further evaluate the performance of *ASCL1*,* LHX8* and *ST6GALNAC5* to detect CIN3 and cervical cancer, we calculated the positivity rates in the referral population for each histology subgroup at the fixed thresholds of 75% and 80% specificity (Table 1). At the 75% specificity thresholds, all samples from women with cervical carcinoma (42/42) tested positive for *ASCL1* and *LHX8*, and 89.9% (38/42) tested positive for *ST6GALNAC5*. Positivity rate of samples from women with CIN3 was 89.4% (42/47) for *ASCL1*, 85.1% (40/47) for *LHX8* and 89.4% (42/47) for *ST6GALNAC5*. At the 80% specificity thresholds, all samples from women with cervical carcinoma tested positive for *ASCL1*, 97.6% (41/42) tested positive for *LHX8* and 90.5% (38/42) tested positive for *ST6GALNAC5*. Positivity rates of samples from women with CIN3 were 89.4% (42/47) for *ASCL1*, 83.0% (39/47) for *LHX8* and 89.4% (42/47) for *ST6GALNAC5*.

**Figure 5:**
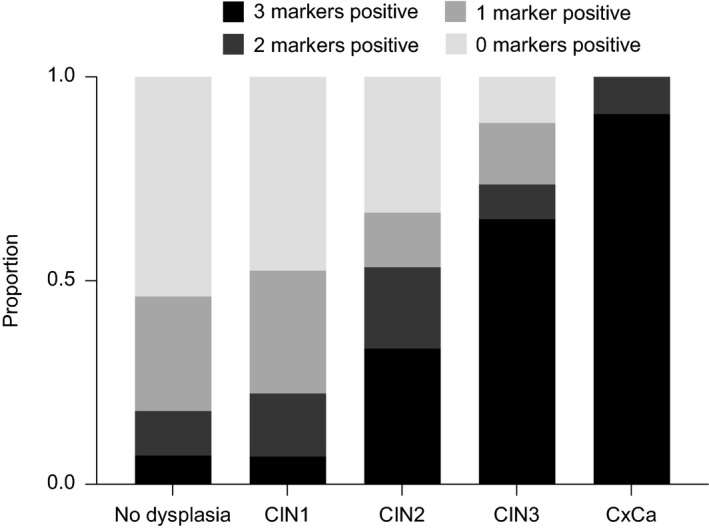
The proportion of hypermethylated *ASCL1*,* LHX8*, and *ST6GALNAC5* genes testing positive in relation to severity of underlying cervical disease. The proportions of samples testing positive for none, one, two or three of the markers within the different histology subgroups (*x*‐axis) from the cohort of WLHIV and the referral population combined are represented on the *y*‐axis. The 75% specificity thresholds were used. CIN, cervical intraepithelial neoplasia; CxCa, cervical carcinomas.

In the referral population and cohort of WLHIV combined, we analysed the proportion of samples testing positive for none, one, two or three of the markers *ASCL1*,* LHX8* and *ST6GALNAC5* within each disease category, at both 75% and 80% specificity thresholds. We found the proportion of samples testing positive for two or three markers to increase with severity of cervical disease at 75% threshold (Figure 5). Similar results were found at the 80% thresholds.

## 4 Discussion

This study reports on primary DNA methylation analysis of host cell genes *ASCL1*,* LHX8* or *ST6GALNAC5* for the detection of cervical cancer and CIN3 in cervical scrapes from WLHIV. In this screening cohort of HIV‐infected women we showed a good CIN3^+^ performance for *ASCL1* and *LHX8* (AUC 0.79 and 0.81 respectively) and a moderate performance for *ST6GALNAC5* (AUC 0.71). At a fixed specificity of 75%, the CIN3^+^ sensitivity of *ASCL1* and *LHX8* was good (72.1% and 73.8% respectively), but the sensitivity of *ST6GALNAC5* was low (55.7%). In line with previously described methylation makers, these markers were highly accurate for the detection of cervical cancer: all carcinomas in this study tested positive for single markers *ASCL1* or *LHX8*, and 89.9% tested positive for *ST6GALNAC5* 21, 22, 34. This indicates that single methylation marker analysis of *ASCL1* or *LHX8* would be an interesting primary cervical screening tool in WLHIV in low‐resource settings, as it detects the majority of CIN3 lesions that need treatment and provides a high reassurance against cervical cancer.

Previous studies described a relationship between HIV and hypermethylation of host cell genes, resulting from an upregulation of DNA methyltransferase expression and activity in HIV‐infected cells 35, 36. This may explain the rather low CIN3^+^ specificity of 49.6% detected in our previous study analysing the methylation marker panel *CADM1, MAL,* and *miR124‐2* 14. Also in this study, the methylation levels for *ASCL1*,* LHX8* and *ST6GALNAC5* were relatively high in control samples of HIV‐infected women compared to HIV‐uninfected women. In contrast to our previous publication, we were able to define hypermethylation positivity thresholds for optimal performance for CIN3^+^ detection in WLHIV and accomplished a relatively high CIN3^+^ specificity, combined with a good sensitivity.

In a setting with limited resources, it is important that women with the highest risk for cervical cancer receive suitable treatment. Early ART initiation and sustained adherence seem to reduce the risk for cervical cancer and its precursor lesions in WLHIV 37, however future studies need to identify which HIV‐infected individuals can be screened less frequently 38. Previous studies have described that methylation marker analysis is specifically sensitive for cervical cancer and cervical lesions caused by a long‐standing (>5 years) persistent hrHPV infection, so called advanced transforming lesions with a high short‐term progression risk. 22, 34, 39, 40 The increase in methylation levels and marker positivity of *ASCL1*,* LHX8* and *ST6GALNAC5* with severity of the underlying lesion, the high sensitivities in an gynaecological outpatient setting and the extremely high methylation levels in cervical carcinomas described in this study, are in line with this concept. This suggests that methylation analysis of these genes identifies lesions with a cancer‐like methylation profile that are in need of treatment.

The simplest approach in low‐resource settings would be to treat all hrHPV positive women, as it yields a high sensitivity 12, 41. Our previous data confirmed the high sensitivity (83.6%) for primary hrHPV testing in WLHIV, but at moderate specificity (67.7%). If such a strategy is implemented without triage testing, a large number of hrHPV positive women (~50%) would receive unnecessary treatment, due to the transient nature of most HPV infections 14. Hence, any triage strategy would require a large amount of secondary tests in a setting with a high HPV prevalence. Subsequent partial hrHPV16/18 genotyping is feasible as it carries the advantages of a molecular test, but with the disadvantage of missing about 30% of non‐HPV16/18 related cervical cancers. Stratification of hrHPV positive women by methylation marker analysis has previously been shown to overcome this issue since it consistently detects all carcinomas 14, 30. This study shows that primary methylation analysis of *ASCL1* or *LHX8* has the same benefit, but without the need for additional triage testing.

An objective and reproducible “see‐and‐treat” strategy for cervical screening in LMIC, which particularly reduces loss to follow‐up, is within reach as methylation assays can be further developed into point‐of‐care tests. Visual inspection with acetic acid, often combined with visual inspection with Lugol's iodine (VILI), is currently recommended by the WHO guidelines for a see‐and‐treat protocol in LMIC 19. Although this approach is cost‐effective and reasonably safe, the subjectivity of the diagnosis influenced by the healthcare provider's experience and environmental conditions, limits the reliability of the technique and leads to under and overtreatment 41, 42, 43, 44, 45. Methylation marker analysis of *ASCL1* or *LHX8* on the other hand, is objective, and is applicable on both cervical scrapes and self‐collected cervical‐vaginal material 26, 27, 46. Although promising, methylation assays are still relatively costly and labour‐intensive, and need further implementation studies. A standardized, easy and robust high‐throughput workflow is needed before implementation in cervical screening can be realised.

Limitations of this study include the cross‐sectional set up as we are still awaiting clinical follow‐up data. Second, to compare hypermethylation levels between HIV seropositive and seronegative women, we enriched the small study group of seronegatives with a Dutch reference population and possible population effects cannot be excluded 47. To differentiate between an HIV effect and a population effect, these comparisons should be repeated with an African HIV uninfected control group. Third, we developed suitable thresholds for scoring methylation marker positivity in the same population as used for the performance analyses of these marker thresholds. A leave‐one‐out cross‐validation approach was used to facilitate these performance analyses. Accordingly, the applicability of *ASCL1* or *LHX8* as primary cervical screening tool requires further validation of this panel in an independent cohort.

This study shows that hypermethylation analysis of *ASCL1* or *LHX8* is a promising method for cervical screening in WLHIV, as well as in an outpatient population. The high sensitivity and high specificity for cervical cancer and CIN3 of these methylation assays, plus their applicability to self‐collected cervical‐vaginal material, make the test a promising screening tool for LMIC and warrants further investigation and development.

## Competing interests

(1) KLR has received speakers fee from Roche diagnostics and MSD; (2) DAMH, PJFS, RDMS and CJLMM are minority shareholders of Self‐screen B.V., a spin‐off company of VUmc; (3) Self‐screen B.V. holds patents related to the work (i.e. hrHPV test and methylation markers for cervical screening); (4) DAMH has been on the speakers bureau of Qiagen and serves occasionally on the scientific advisory boards of Pfizer and Bristol‐Myers Squibb; (5) PJFS has been on the speakers bureau of Roche diagnostics, Gen‐Probe, Abbott, Qiagen and Seegene and has been a consultant for Crucell B.V.; (6) CJLMM has received speakers fee from GSK, Qiagen, SPMSD/Merck, Roche diagnostics, Menarini and Seegene, served occasionally on the scientific advisory board (expert meeting) of GSK, Qiagen, SPMSD/Merck, Roche and Genticel and has been by occasion consultant for Qiagen and Genticel; (7) CJLMM has a very small number of shares of Qiagen and holds minority stock in Self‐Screen B.V. Until April 2016 he was minority shareholder of Diassay B.V., and until 2014 he held a small number of certificates of shares in Delphi Biosciences; (8) CJLMM is part‐time director of Self‐screen B.V. since September 2017; (9) all other authors declare that they have no conflict of interest.

## Authors' contributions

GD and CJLMM are principal investigators of the study. WWK, MZ, PJF, GD and CJLMM have set up the trial. WWK, MZ, KLR, WV, PJFS, DAMH, RDMS, GD and CJLMM were involved in data collection. WWK and PWN performed the statistical analysis. WWK and MZ managed the database. WWK, MZ, DAMH, RDMS and CJLMM drafted the manuscript. All authors critically reviewed the manuscript and approved the final version. All authors had full access to all of the data in the study and can take responsibility for the integrity of the data and the accuracy of the data analysis and believe that the manuscript represents honest work. CJLMM affirms that the manuscript is an honest, accurate and transparent account of the study being reported; that no important aspects of the study have been omitted; and that any discrepancies from the study as planned have been explained.
